# Altered Phosphorylation of the Proteasome Subunit Rpt6 Has Minimal Impact on Synaptic Plasticity and Learning

**DOI:** 10.1523/ENEURO.0073-20.2021

**Published:** 2021-05-04

**Authors:** Samantha L. Scudder, Frankie R. Gonzales, Kristin K. Howell, Ivar S. Stein, Lara E. Dozier, Stephan G. Anagnostaras, Karen Zito, Gentry N. Patrick

**Affiliations:** 1 Section of Neurobiology, Division of Biological Sciences, University of California San Diego, La Jolla, CA 92093-0347; 2 Molecular Cognition Laboratory, Department of Psychology, University of California San Diego, La Jolla, CA 92093-0109; 3 Center for Neuroscience, University of California, Davis, CA 95618-4859

**Keywords:** degradation, dendritic spines, LTP, plasticity, proteasome, Rpt6

## Abstract

Dynamic control of protein degradation via the ubiquitin proteasome system (UPS) is thought to play a crucial role in neuronal function and synaptic plasticity. The proteasome subunit Rpt6, an AAA ATPase subunit of the 19S regulatory particle (RP), has emerged as an important site for regulation of 26S proteasome function in neurons. Phosphorylation of Rpt6 on serine 120 (S120) can stimulate the catalytic rate of substrate degradation by the 26S proteasome and this site is targeted by the plasticity-related kinase Ca^2+^/calmodulin-dependent kinase II (CaMKII), making it an attractive candidate for regulation of proteasome function in neurons. Several *in vitro* studies have shown that altered Rpt6 S120 phosphorylation can affect the structure and function of synapses. To evaluate the importance of Rpt6 S120 phosphorylation *in vivo*, we created two mouse models which feature mutations at S120 that block or mimic phosphorylation at this site. We find that peptidase and ATPase activities are upregulated in the phospho-mimetic mutant and downregulated in the phospho-dead mutant [S120 mutated to aspartic acid (S120D) or alanine (S120A), respectively]. Surprisingly, these mutations had no effect on basal synaptic transmission, long-term potentiation (LTP), and dendritic spine dynamics and density in the hippocampus. Furthermore, these mutants displayed no deficits in cued and contextual fear memory. Thus, in a mouse model that blocks or mimics phosphorylation at this site, either compensatory mechanisms negate these effects, or small variations in proteasome activity are not enough to induce significant changes in synaptic structure, plasticity, or behavior.

## Significance Statement

Several studies have shown that phosphorylation of the proteasome subunit Rpt6 at serine 120 (S120) is an important regulatory mechanism for proteasome-dependent control of synapses and plasticity. We generated phospho-dead and phospho-mimetic knock-in (KI) mice to directly evaluate the role of this site. While we observed expected changes in proteasome activity, we surprisingly did not observe significant changes in basal synaptic transmission, hippocampal long-term potentiation (LTP), dendritic spine outgrowth, or behavior. This suggests that neurons may use additional mechanisms to compensate for alterations in proteasome function to mediate the tight control of the synaptic proteome required for proper synaptic structure, function, and plasticity.

## Introduction

Synaptic plasticity, which is fundamental for learning and memory, is mediated by both morphologic and functional modifications to synapses. Furthermore, there is a growing body of evidence which suggests that impairments in synaptic plasticity contribute to neuropsychiatric disorders and neurodegenerative diseases. Emerging evidence has shown that local protein synthesis and degradation are crucial for the remodeling of synapses (Alvarez-Castelao and Schuman, 2015). Protein homeostasis, the balance between protein synthesis and degradation, is critical for maintaining cell health and viability. The ubiquitin proteasome system (UPS) is a major pathway through which proteins are broken down and recycled in eukaryotic cells, making it pivotal in their development, maintenance, and survival. The 26S proteasome is a multisubunit complex which breaks down proteins targeted for degradation (Hershko and Ciechanover, 1998), and consists of a 20S catalytic core and a 19S regulatory particle (RP). The 19S RP includes a six subunit hexameric ring of AAA ATPases (Rpt1-6) which serve as the interface with the α subunits of the core particle (CP) and which provide the mechanical energy necessary to deubiquitinate substrates and move them into the catalytic core (Lander et al., 2012; Matyskiela et al., 2013). Protein homeostasis is particularly important in the CNS, as rapid response to external cues, maintaining and remodeling synaptic connections, and constantly adjusting the protein composition of presynaptic and postsynaptic compartments is critical in synaptic function and dysfunction (Ding et al., 2006; Graham and Liu, 2017).

In mammals, studies have shown that proteasome inhibition alters basal synaptic transmission, long-term potentiation (LTP), and long-term depression (LTD; Deng and Lei, 2007; Fonseca et al., 2006; Hou et al., 2006; Karpova et al., 2006). Interestingly, there appears to be a strict balance between new protein synthesis and protein degradation via the proteasome. While the application of protein synthesis inhibitors or proteasome inhibitors alone block LTP, the application of these drugs together occludes the effect of each other as LTP is unaltered (Fonseca et al., 2006). These studies highlight the idea that the relative concentration of key synaptic proteins can be rate-limiting for long-lasting changes in synaptic efficacy. We have previously shown that the blockade of action potentials with tetrodotoxin or upregulation of activity with bicuculline inhibit and increase proteasome peptidase activity, respectively (Djakovic et al., 2009). Ca^2+^/calmodulin-dependent kinase IIα (CaMKIIα) has been directly linked to this phenomenon, as it was demonstrated that CaMKIIα acts as a scaffold protein necessary for proteasome translocation into spines. CaMKIIα also stimulates proteasome peptidase activity through activity-dependent phosphorylation of serine 120 (S120) on Rpt6, an ATPase subunit on the 19S RP of the proteasome (Bingol and Schuman, 2006; Djakovic et al., 2009, 2012; Bingol et al., 2010). In subsequent *in vitro* investigations, we showed that homeostatic scaling of synaptic strength induced by chronic changes in synaptic activity is impaired by altered Rpt6 S120 phosphorylation (Djakovic et al., 2012). We also demonstrated that the acute proteasomal inhibition or ectopic expression of an Rpt6 S120A phospho-dead mutant blocks activity-dependent new spine generation (Hamilton et al., 2012). Furthermore, phosphorylation of S120 is increased during fear conditioning, and CaMKIIα-dependent regulation of proteasome phosphorylation and activity promotes memory consolidation (Jarome et al., 2013, 2016). Together, these data suggest that CaMKIIα-dependent phosphorylation of Rpt6 at S120 may be an important regulatory mechanism for proteasome-dependent control of synaptic remodeling in various plasticity and behavioral paradigms.

We sought to further investigate the importance of Rpt6 phosphorylation and proteasome function in neurons. Historically, proteasome inhibitors have been used to understand the role of the UPS in synaptic plasticity and behavior. However, they are quite toxic to cells and therefore have a limited utility. Many loss**-**of**-**function mutations in proteasome subunits cause lethality in various organisms, therefore the need for more subtle mutant models which partially alter proteasome function is quite high. We have generated two novel knock-in (KI) mouse models which eliminate Rpt6 S120 phosphorylation dynamics by mutating Rpt6 S120 to either alanine (S120A), which blocks phosphorylation, or to aspartate (S120D), which mimics phosphorylation because of its negatively charged carboxyl group. With these mouse models, we set out to examine the effects of altered Rpt6 S120 phosphorylation in the brain.

We found that affinity-purified proteasomes from Rpt6 S120A and S120D mutant mice exhibit alterations in kinetic rate of ATP hydrolysis and substrate degradation when compared with wild-type (WT) littermates. Interestingly, however, we did not observe any significant changes in basal synaptic transmission and hippocampal LTP. In addition, spine density and outgrowth dynamics were unaltered in Rpt6 S120A mutants. Furthermore, fear memory, locomotor behavior, and anxiety-related behaviors were normal in both Rpt6 S120A and S120D mutants. These findings are in stark contrast to previous data which showed significant differences in synapse strength, synaptic scaling, and activity-dependent spine outgrowth (Djakovic et al., 2009, 2012; Hamilton et al., 2012) This suggests that in neurons additional mechanisms may exist to compensate for alterations in proteasome function and mediate the tight control of the synaptic proteome required for proper changes in synaptic structure, synaptic function, and behavior.

## Materials and Methods

### Generation of Rpt6 S120A and S120D KI mice

We generated Rpt6 phospho-mimetic (S120 to aspartic acid; S120D), and phospho-dead (S120 to alanine; S120A) KI mice (inGenious Targeting Laboratory). The exact same targeting strategy for Rpt6 S120D mutant mice was previously described in Gonzales et al. (2018). The targeting vectors were linearized and transfected by electroporation into BA1 (C57Bl/6 × 129/SvEv; Hybrid) embryonic stem cells. Selected clones were expanded for Southern blot analysis to identify recombinant ES clones (data not shown). The ES clones were microinjected into C57BL/6 blastocysts. After germline transmission, the Neo cassette was removed by mating to C57BL/6 FLP mice. Tail DNA was analyzed by PCR to identify heterozygous mice and verify deletion of the Neo cassette. Mutant heterozygous mice were backcrossed to C57BL/6. By visual inspection, Rpt6 S120D and S120A homozygous mutants (confirmed by PCR and sequencing), obtained by crossing heterozygous mutants, displayed normal body size, feeding, and mating behaviors. The intercross of heterozygotes resulted in production of WT, heterozygous, and homozygous offspring at the expected 1:2:1 Mendelian ratio. All procedures were approved by the local Institutional Animal Care and Use Committee and compliant with the National Research Council Guide.

### Antibodies and reagents

Anti-20S core α subunits monoclonal mouse antibody (mAb MCP231) and Rpt6 regulatory subunit monoclonal mouse antibody (mAb p45-110) were purchased from Enzo Life Sciences. Rabbit PSD-95 antibody was purchased from Calbiochem. Custom rabbit (pAb; clone #07) anti-Rpt6 phospho-specific antibody for S120 (pS120) was previously generated commercially (ProSci) against a synthetic phosphorylated peptide (Djakovic et al., 2012). Fluorescent and HRP-conjugated secondary antibodies were purchased from Thermo Fisher Scientific. N-succinyl-Leu-Leu-Val-Tyr-7-amino-4-methylcoumarin (Suc-LLVY-AMC) substrate was used (BACHEM). GST-Ubl and His-UIM constructs were a gift from the Alfred Goldberg lab. Malachite Green and Adenosine triphosphate were purchased from Sigma-Aldrich. PhosSTOP kinase/phosphatase inhibitor and cOmplete protease inhibitors were purchased from Roche. Picrotoxin was purchased from Tocris.

### Proteasome purification

Purification was conducted as previously described (Besche and Goldberg, 2012), with some modifications. Whole-brain lysates were dounce homogenized in affinity purification buffer [APB; 25 mm HEPES–KOH (pH 7.4), 10% glycerol, 5 mm MgCl_2_, 1 mm ATP, and 1 mm DTT] and lysates cleared by ultra-centrifugation at 100,000 × *g* for 60 min. Lysate was incubated with purified GST-Ubl recombinant protein at a concentration of 0.2 mg/ml, then GSH-agarose beads were added and incubated for 2 h at 4°C. The slurry was loaded onto a 20-ml column and washed twice with 10-ml APB. Proteasomes were eluted with purified UIM (2 mg/ml) in two incubations of 250 μl each. The resulting purified proteasomes were then measured for protein concentration, aliquoted, and frozen at −80°C. Trypsinized samples were additionally subjected to mass spectrometry analysis to further confirm mutant Rpt6 incorporation and 26S proteasome composition and interactions.

### Peptidase and ATPase assays

Peptidase assays were conducted according to methods previously described (Kisselev and Goldberg, 2005). For peptidase assays, purified proteasomes were mixed with assay buffer [50 mm HEPES (pH 7.8), 10 mm NaCl, 1.5 mm MgCl_2_, 1 mm EDTA, 1 mm EGTA, 250 mm sucrose, 5 mm DTT, and 2 mm ATP] and suc-LLVY-AMC substrate, 100 μl was loaded into a Costar black 96 well microassay plate (in triplicate), and the kinetic rate of cleavage was monitored by the increase in fluorescence (excitation: 360 nm; emission: 465 nm) at 37°C with a microplate fluorimeter (PerkinElmer HTS7000 Plus) every 30 s for 2 h. The slope of the plot was calculated as the relative kinetic rate of substrate cleavage by the proteasome. The Malachite green assay, a colorimetric assay used to measure the evolution of inorganic phosphate from a mixture of pure proteasomes and ATP, was adapted from previous studies (Lanzetta et al., 1979) to quantify ATP hydrolysis. The assay was conducted in triplicate on clear Costar 96 well plates, and the absorption at 660 nm was measured via plate reader spectrophotometer at different time points. The slope of the graph was calculated as the kinetic rate of ATP hydrolysis.

### Western blotting and native gel assays

Equal amounts of purified proteasomes were resolved by SDS-PAGE, then transferred onto nitrocellulose membranes. Membranes were then probed for proteasome α-subunits, total Rpt6, and phospho-Rpt6. In-gel activity assays were performed by loading equal amounts of purified proteasomes onto native gradient gels (4–12%) and resolving overnight at 4°C. The gel was then soaked in developing buffer [50 mm Tris-HCl (pH 7.4), 5 mm MgCl_2_, 0.5 mm EDTA, and 1 mm ATP] and supplemented with 50 μm suc-LLVY-AMC substrate for 30 min at room temperature. The gel was then imaged in a Protein Simple FluorChem E imaging system, resulting in fluorescent bands. Gels were then transferred to nitrocellulose and probed for 20S core subunits, Rpt6, and phospho-Rpt6 and then secondary HRP-conjugated antibodies. Resulting blots were digitized by scanning films and band intensities were quantified using NIH ImageJ.

### Slice electrophysiology

Acute hippocampal slices were prepared from three- to eight-week-old mice with experimenter blind to genotype. Mice were anesthetized with isoflurane before decapitation and brain extraction into ice-cold sucrose-containing artificial CSF (ACSF; 83 mm NaCl, 2.5 mm KCl, 1 mm NaH_2_PO_4_, 26.2 mm NaHCO_3_, 22 mm glucose, 72 mm sucrose, 0.5 mm CaCl_2_, and 3.3 mm MgSO_4_). Tissue was sliced coronally into 350 μm slices using a Leica VT1200 vibratome. Slices were recovered in standard ACSF (119 mm NaCl, 5 mm KCl, 1 mm NaH_2_PO_4_, 26 mm NaHCO_3_, 11 mm glucose, 2 mm CaCl_2_, and 1 mm MgSO_4_) at 34°C for 30 min and at room temperature for at least 30 min before recordings. Slices were transferred to a submerged recording chamber and perfused with room-temperature (basal transmission experiments) or 30°C (potentiation experiments) oxygenated ACSF (with 100 μm picrotoxin).

A cluster stimulating electrode (FHC) was placed in the stratum radiatum of the CA1 region and current or voltage was injected using an ISO-Flex stimulus isolator (A.M.P.I.) triggered by a Clampex 10.3 (Molecular Devices) or custom IGOR Pro protocol. Recording electrodes were generated from thin-walled capillary tubing (Warner Instruments) using a horizontal pipette puller (P-97 Flaming/Brown Micropipette Puller, Sutter Instruments), resulting in a resistance of 1–3 MΩ, and pipettes were filled with ACSF. The recording electrode was placed 200–300 μm away from the stimulating electrode in the stratum radiatum, along a pathway parallel to the CA1 pyramidal layer. A second stimulating electrode was placed on the opposite side of the recording electrode to serve as a control pathway in LTP experiments. Field responses were recorded using an Axopatch 200B amplifier (Molecular Devices) and digitized using a Digidata 1322 digitizer (Molecular Devices), and signals were acquired using Clampex 10.3 or IGOR Pro.

Field excitatory postsynaptic potentials (fEPSPs) were evoked using 100-μs pulses. Input-output relationships were determined by recording fEPSPs at a variety of stimulus intensities ranging from 30 to 130 μA and averaging five traces per intensity. Paired pulse facilitation was examined by generating pulses at variable separations (400, 200, 150, 100, and 50 ms) and taking the ratio of the second fEPSP amplitude to the first, with three recordings averaged for each separation. To study LTP, a stimulus intensity that produces a response of 0.2–0.4 mV was administered once every 15 s until a stable baseline of 15 min was reached. LTP was induced in one pathway using one set or four sets of 1-s 100-Hz trains separated by 20 s and post-LTP responses were recorded from both control and experimental pathways for 40–60 min after potentiation.

### Behavioral assessment

#### Fear conditioning

Mice were tested in individual conditioning chambers. The VideoFreeze system (Med Associates) was used to assess behavior as described previously (Anagnostaras et al., 2010; Carmack et al., 2014). Training consisted of one 10-min session. Mice were placed in chambers and baseline activity was assessed for 2 min. Mice then received three tone-shock pairings beginning at minutes 2, 3, and 4. Tone-shock pairings consisted of a 30-s tone (2.8 kHz, 90 dB) that co-terminated with a 2-s scrambled AC foot shock (0.75 mA, RMS). Twenty-four hours post-training, mice were returned to the training context. Once mice were placed in the chambers, freezing was measured for 5 min to assess fear to the context. Twenty-four hours after the context test a 5 min tone test was conducted to assess cued memory. The context was altered on multiple dimensions. White acrylic sheets were placed over the grid floors, a black plastic triangular insert was used to alter wall shape, and chambers were cleaned and scented with a 5% vinegar solution. Near-infrared light was used, in the absence of white light, to create a dark environment. The test consisted of a 2-min baseline period followed by the presentation of three 30-s tones (2.8 kHz, 90 dBA) at minutes 2, 3, and 4. The tones matched those used during training.

#### Elevated plus maze

The plus maze (MED Associates) had two open and two enclosed arms (6.5 × 36 cm each) joined at a center hub (6.5 × 6.5 cm) elevated 74 cm from the ground. Testing lasted 5 min in dim light. The floor of the maze had near-infrared backlighting invisible to the mice to provide video contrast. Mice were tracked using a camera and video tracking software (Panlab Smart 3.0, Harvard Apparatus). Time spent in each arm and total distance traveled were recorded.

### Spine density and immunohistochemistry

Adolescent [postnatal day (P)21–P28] homozygous Rpt6 S120A and corresponding WT mice were given an anesthetic analgesic solution of ketamine and xylazine before intracardial perfusion with 0.9% saline, followed by 4% paraformaldehyde (PFA) fixative solution (20 ml 0.2 m PB, 10 ml ddH_2_O, and 10 ml 16% PFA). Brains were postfixed for 1 h, then 100-μm coronal sections were cut using a Vibratome. Penetrating microelectrodes were pulled from borosilicate capillary glass with filament (1-mm outer diameter/−0.58-mm inner diameter) and then backfilled with KCl (200 mm) and Alexa Fluor 594 hydrazide (10 mm) solution (Invitrogen). Using micromanipulation and visual guidance, CA1 neurons were filled via iontophoresis. Sections were postfixed for 10 min before mounting with Aqua Polymount (Polysciences Inc). Confocal microscopy was used to image secondary apical dendrites for analysis. The density, length, and width of dendritic protrusions were measured via Z-stacks in ImageJ using custom macros blind to genotype. For Rpt6 and PSD-95 staining, dissociated hippocampal cultures from S120D and WT littermates were used at 21–24 d *in vitro* (DIV). Neurons were infected with a *Sindbis virus* containing genetic material for green fluorescent protein (GFP) for 14–24 h to fill dendrites to allow for selection and analysis. Cells were fixed using a 4% PFA fixative solution, permeabilized with 0.2% Triton X-100, blocked in 5% bovine serum albumin, probed for total Rpt6 and PSD-95 during overnight incubation at 4°C, then incubated with fluorescent-conjugated secondary antibodies at room temperature. Confocal microscopy was used to image up to two secondary apical dendrites per cell for analysis, and custom macros in ImageJ were used to process and analyze images. Experimenters (blind to genotype) drew regions of interest (ROIs) around all visible dendritic spines to determine the mean fluorescence of Rpt6 and PSD-95 in spine compartments. Extent of colocalization of these two proteins was determined using Pearson’s correlation.

### Preparation and transfection of dissociated and organotypic slice cultures

Organotypic hippocampal slices were prepared from litter matched P6–P8 WT and S120A KI mice of both sexes, as previously described (Stoppini et al., 1991). The cultures were transfected 1–2 d before imaging via biolistic gene transfer (180 psi), as previously described (Woods and Zito, 2008). 10–15 μg of EGFP (Clontech) was coated onto 6–8 mg of 1.6-μm gold beads. Dissociated cultures from S120D and WT mice were generated from the hippocampi of P1 pups of either sex. Cells were plated at a density of 45,000 cells/cm^2^ onto poly-D-lysine-coated and laminin-coated coverslips and were maintained in B27-supplemented Neurobasal media (Invitrogen) until at least 21 DIV.

### Time-lapse imaging and image analysis for spine dynamics

EGFP-transfected CA1 pyramidal neurons (7–9 DIV) at depths of 10–50 μm were imaged using a custom two-photon microscope (Woods et al., 2011) controlled with ScanImage (Pologruto et al., 2003). Slices were imaged at 29°C in recirculating ACSF containing the following: 127 mm NaCl, 25 mm NaHCO_3_, 25 mm D-glucose, 2.5 mm KCl, 1.25 mm NaH_2_PO_4_, 1 mm MgCl_2_, and 2 mm CaCl_2_, aerated with 95%O_2_/5%CO_2_. For each neuron, image stacks (512 × 512 pixels; 0.035 μm/pixel) with 1-μm z-steps were collected from five to six segments of secondary dendrites (apical and basal) at 15-min intervals. To compare rates of activity-induced new spine formation in WT versus S120A KI mice, slices were treated with 30 μm bicuculline (Tocris, 1,000× aqueous stock) or vehicle. All shown images are maximum projections of three-dimensional (3D) image stacks after applying a median filter (3 × 3) to the raw image data. Spine formation rates were blindly analyzed in 3D using custom software in MATLAB.

### Statistical analyses

For comparison of two groups, we used a standard *t* test. For groups of three or more, we performed a one-way ANOVA with Tukey’s *post hoc* multiple comparison test or two-way ANOVA with Bonferroni’s *post hoc* multiple comparison test. Significance threshold was set at *p* < 0.05. Behavioral data were analyzed using multivariate or univariate ANOVAs. Significance was set at level *p *<* *0.05 (SPSS Statistics Desktop, V22.0 or GraphPad Prism). Graphs depict mean ± SEM.

## Results

### Generation, purification, and analysis of Rpt6 S120A and S120D mutant proteasome composition and activity

In order to evaluate the functional relevance of Rpt6 S120 phosphorylation in the brain and at synapses, we generated two novel mouse lines by mutating Rpt6 S120 to either alanine (S120A), which blocks phosphorylation, or to aspartate (S120D), which mimics phosphorylation because of its negatively charged carboxyl group. The targeting strategy for Rpt6 S120D mutant mice was previously described in Gonzales et al. (2018). Rpt6 S120A mutant mice were made at the same time using the same targeting strategy to create the phospho-ablated mutant. Codon 120 in exon 5 of the *PSMC5 (Rpt6)* gene was mutated from AGC to GCC (S120 to ala; Gonzales et al., 2018; Extended Data Fig. 1-1; see also Materials and Methods). By visual inspection, Rpt6 S120A and Rpt6 S120D homozygous mutants displayed normal body size, feeding, and mating behaviors. In addition, there were no obvious gross brain anatomic differences observed (Extended Data Fig. 1-1).

**Figure 1.**
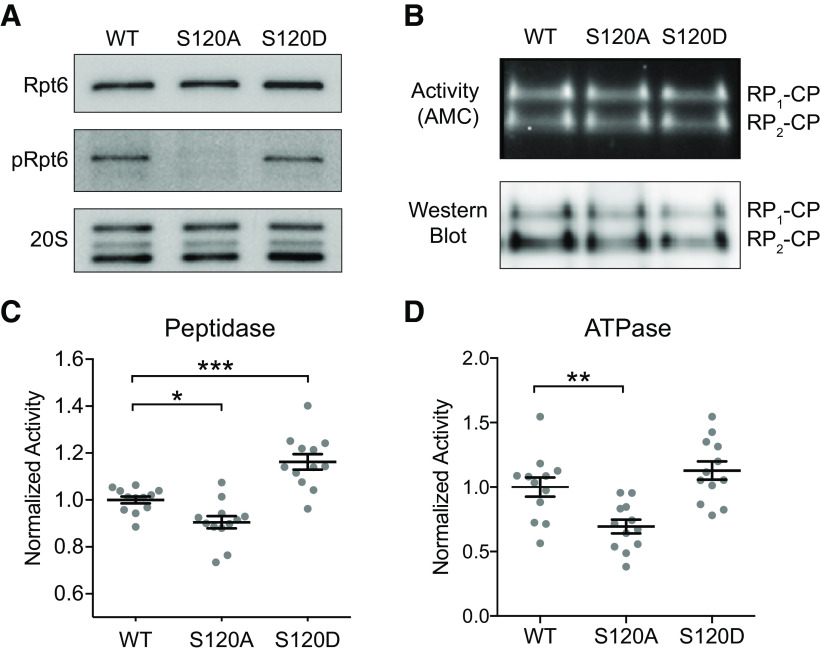
Proteasome protein degradation and ATP hydrolysis kinetics are significantly altered in Rpt6 S120A and S120D KI mice. ***A***, Representative Western blot analysis of proteasomes purified using Ubl/UIM method, and verification of phospho-Rpt6 antibody specificity to WT Rpt6 with expected cross reaction with S120D Rpt6 (performed for each purification). ***B***, Native gel fluorescent activity assay and tandem Western blotting with two bands representing singly and doubly capped proteasomes (performed for each purification). ***C***, Fluorescent peptidase activity assay; S120A has significantly decreased activity (*p* = 0.04, *post hoc* Tukey, *n* = 12), S120D shows increased activity (*p* < 0.001, *post hoc* Tukey, *n* = 12), and ANOVA indicates significant difference within the group (*F* = 25.26, *p* < 0.001). ***D***, Malachite green ATPase assay; S120A displays significantly lower activity (*p* = 0.007, *post hoc* Tukey, *n* = 12), while S120D shows increased activity (*p* = 0.37, *post hoc* Tukey, *n* = 12), and the one-way ANOVA indicates significant difference within the group (*F* = 11.21, *p* < 0.001). *p < 0.05; **p < 0.01; ***p < 0.001.

10.1523/ENEURO.0073-20.2021.f1-1 Extended Data Figure 1-1 Generation of Rpt6 S120A and S120D KI mice. ***A***, The original targeting strategy for Rpt6 S120D mutant mice was previously described in Gonzales et al. (2018). Rpt6 S120A mutant mice were made at the same time using the same targeting strategy to create the phospho-ablated mutant. Codon 120 in exon 5 of the *PSMC5 (Rpt6)* gene was mutated from AGC to GCC (S120 to ala; Gonzales et al., 2018; see also Materials and Methods). ***A***, Tail genomic DNA was analyzed by PCR screening for genotyping and to verify deletion of the Neo cassette. ***B***, Representative electropherograms confirming the presence of the mutation in homozygous Rpt6 S120A and S120D mutant male mice. ***C***, Representative images of Nissl-stained fixed whole-brain coronal sections (with higher magnification of the hippocampus) of 60-d-old mice. Download Figure 1-1, PDF file.

We evaluated proteasome peptidase and ATPase activity of proteasomes purified from Rpt6 WT and mutant mice. The gentle affinity purification protocol we used more efficiently maintains interactions between the regulatory and catalytic particles of the proteasome, as well as those between the proteasome and its interacting proteins (Besche and Goldberg, 2012). In order to verify efficient purification of WT and mutant proteasomes, we resolved the purification products on SDS-PAGE gels. Western blottings showed high concentrations of 20S subunits as well as total Rpt6. When we probed for phospho-Rpt6 using our affinity purified phospho-Rpt6 antibody, S120A mutants showed little to no cross-reactivity, while S120D showed marginal cross-reactivity, as expected (Fig. 1*A*). In order to evaluate composition and functionality of our purified proteasomes, we ran the purification products on a native gel and performed an in-gel fluorescence assay using the LLVY-AMC substrate. After electrophoresis, we bathed the gel in a buffer containing the substrate and imaged it on a Protein Simple UV imager, rendering bands for both single and doubly capped proteasomes (Fig. 1*B*), verifying that our proteasomes were intact and fully functional following purification. We further confirmed the Rpt6 incorporation and composition of purified proteasomes by mass spectrometry analysis (data not shown).

In order to quantitatively evaluate proteasome activity, we used kinetics assays to measure peptidase and ATPase activities to determine whether they exhibit the same differences as they did in cultures. We found that Rpt6 S120A phospho-dead mutants showed significantly reduced peptidase activity as compared with WT (*p* = 0.04; *n* = 12), while Rpt6 S120D phospho-mimetic mutants exhibit significantly increased peptidase activity (*p* < 0.001; *n* = 12; Fig. 1*C*).

Rpt6 is one of six ATPase subunits in the 19S RP. These subunits hydrolyze ATP, providing the mechanical energy necessary to unfold target substrates and move them inside the catalytic core (Yedidi et al., 2017). We therefore investigated the ATPase activities of our purified WT and mutant proteasomes. Indeed, we found that the S120A mutant displayed a reduced rate of ATPase activity compared with WT (*p* = 0.007; *n* = 12), and the S120D mutant showed a slight but statistically insignificant increase in activity (*p* = 0.37; *n* = 12; Fig. 1*D*).

### Elimination of phosphorylation dynamics do not affect basal synaptic transmission nor LTP paradigms of synaptic plasticity

**Figure 2.**
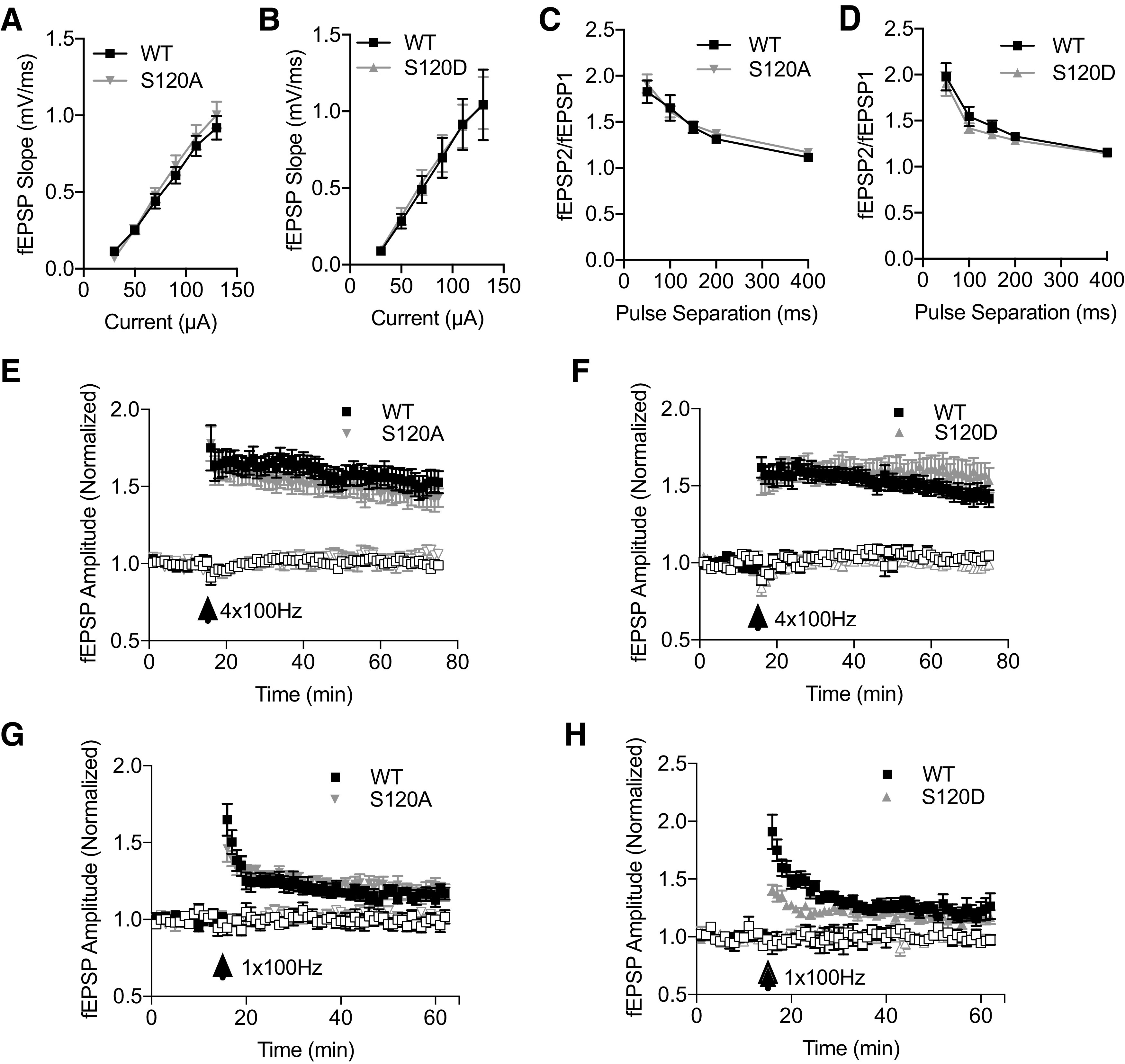
Basal synaptic transmission and LTP are unaltered in Rpt6 S120A and S120D KI mice. ***A***, Input/output curve in slices from WT (*n* = 12 slices from 7 mice) and S120A mice (*n* = 17 slices from 7 mice), depicting increasing fEPSP responses to increased amplitude of Schaffer collateral stimulation with no difference between groups (two-way ANOVA, interaction: *F*_(5,147)_ = 0.21; *p* = 0.96). ***B***, Input/output curve in acute hippocampal slices from WT (*n* = 11 slices from 7 mice) and S120D mice (*n* = 11 slices from 7 mice), with no differences between groups (two-way ANOVA, interaction: *F*_(5,115)_ = 0.02; *p* = 0.99). ***C***, Two successive stimuli with short separation delivered to the Schaffer collateral of acute hippocampal slices leads to enhancement of fEPSP amplitude (paired pulse facilitation) in WT (*n* = 15 slices from 7 mice) and S120A mice (*n* = 16 slices from 7 mice), with similar facilitation in both groups (two-way ANOVA, interaction: *F*_(4,145)_ = 0.22; *p* = 0.93). ***D***, Paired pulse facilitation in WT (*n* = 10 slices from 7 mice) and S120D mice (*n* = 9 slices from 7 mice), showing no differences between groups (two-way ANOVA, interaction: *F*_(4,85)_ = 0.15; *p* = 0.96). ***E***, Delivery of four 1-s trains (20 s apart) of 100-Hz stimulation causes potentiation of fEPSP amplitude in acute hippocampal slices from WT (*n* = 9 slices from 7 mice) and S120A mice (*n* = 6 slices from 6 mice). ***F***, LTP induction in response to four 1-s 100-Hz trains in WT (*n* = 9 slices from 9 mice) and S120D mice (*n* = 10 slices from 10 mice). ***G***, LTP induction in response to a single 1-s 100-Hz train in slices from WT (*n* = 6 slices from 4 mice) and S120A animals (*n* = 7 slices from 4 mice). ***H***, LTP induction in response to a single 1-s 100-Hz train in WT (*n* = 4 slices from 4 mice) and S120D animals (*n* = 6 slices from 4 mice). For ***E–H***, filled squares and triangles indicate stimulated (LTP) pathway while open symbols denote control pathway. Graphs depict mean ± SEM for each current intensity, pulse separation, or time point.

We first assessed whether Rpt6 mutations affect basal synaptic transmission using Schaffer collateral (CA3–CA1) stimulation to generate fEPSPs in acute hippocampal slices from mutant and WT mice. We observed that slices from both S120A and S120D animals display a normal input-output relationship when compared with slices obtained from age-matched WT mice (S120A vs WT: *p* = 0.96, *n* = 12 slices from 7 mice and 17 slices from 7 mice; S120D vs WT: *p* = 0.99, *n* = 11 slices from 7 mice and 11 slices from 7 mice; Fig. 2*A*,*B*). This indicates that the hippocampal circuit has developed normally and has roughly the same amount of synaptic connectivity as a WT hippocampus. Thus, these specific mutations to S120 do not obviously impact the formation and maintenance of excitatory synapses in the hippocampus, and do not appear to strongly impact synaptic strength at these synapses. To assess whether presynaptic release mechanisms were altered in S120A and S120D mice, we next used a paired pulse facilitation assay. By stimulating Schaffer collateral axons twice at varying pulse separations, we revealed strong facilitation of fEPSPs in WT slices at small separations (50, 100 ms). S120A and S120D slices displayed identical levels of facilitation (S120A vs WT: *p* = 0.93, *n* = 15 slices from 7 mice and 16 slices from 7 mice; S120D vs WT: *p* = 0.96, *n* = 10 slices from 7 mice and 9 slice from 7 mice), indicating that presynaptic neurotransmitter release is intact and has normal responses to increased presynaptic calcium (Fig. 2*C*,*D*).

Since previous work with proteasome inhibitors pointed to an important role for these complexes in mediating LTP, we next sought to determine whether our Rpt6 mutations would affect this plasticity paradigm. We generated acute hippocampal slices from young (P21–P27) age-matched mice and induced LTP in the CA3–CA1 pathway using four trains of 1-s 100-Hz stimulation. We observed that hippocampal slices from S120A (normalized amp. during last 5 min: WT = 1.52 ± 0.07; S120A = 1.43 ± 0.07; *p* = 0.38; *n* = 9 slices from 7 mice and 6 slices from 6 mice) and S120D animals (normalized amp.: WT = 1.44 ± 0.05; S120D = 1.57 ± 0.08; *p* = 0.21; *n* = 9 slices from 9 mice and 10 slices from 10 mice) undergo normal levels of fEPSP potentiation compared with WT mice (Fig. 2*E*,*F*).

Because of previous studies suggesting that proteasome activity can modulate LTP induction thresholds (Dong et al., 2008), we next used a weaker stimulation paradigm, instead stimulating with a single 1-s 100-Hz train. While both S120A (normalized amp. during last 5 min: WT = 1.16 ± 0.03; S120A = 1.19 ± 0.05; *p* = 0.66; *n* = 6 slices from four mice and 7 slices from four mice) and S120D (normalized amp. during last 5 min: WT = 1.22 ± 0.08; S120D = 1.17 ± 0.05; *p* = 0.56; *n* = 4 slices from four mice and 6 slices from four mice) mutants displayed normal levels of slight potentiation 40–45 min after LTP induction (Fig. 2*G*,*H*), we did observe a suppression of post-tetanic potentiation and early LTP in S120D animals (normalized amp. during first 5 min: WT = 1.66 ± 0.08; S120D = 1.33 ± 0.04; *p* = 0.004; Fig. 2*G*). However, since potentiation eventually reached WT levels, it appears that neither mutation has a strong effect on LTP.

### Spine density, outgrowth dynamics, and proteasome distribution are not altered in KI mice

**Figure 3.**
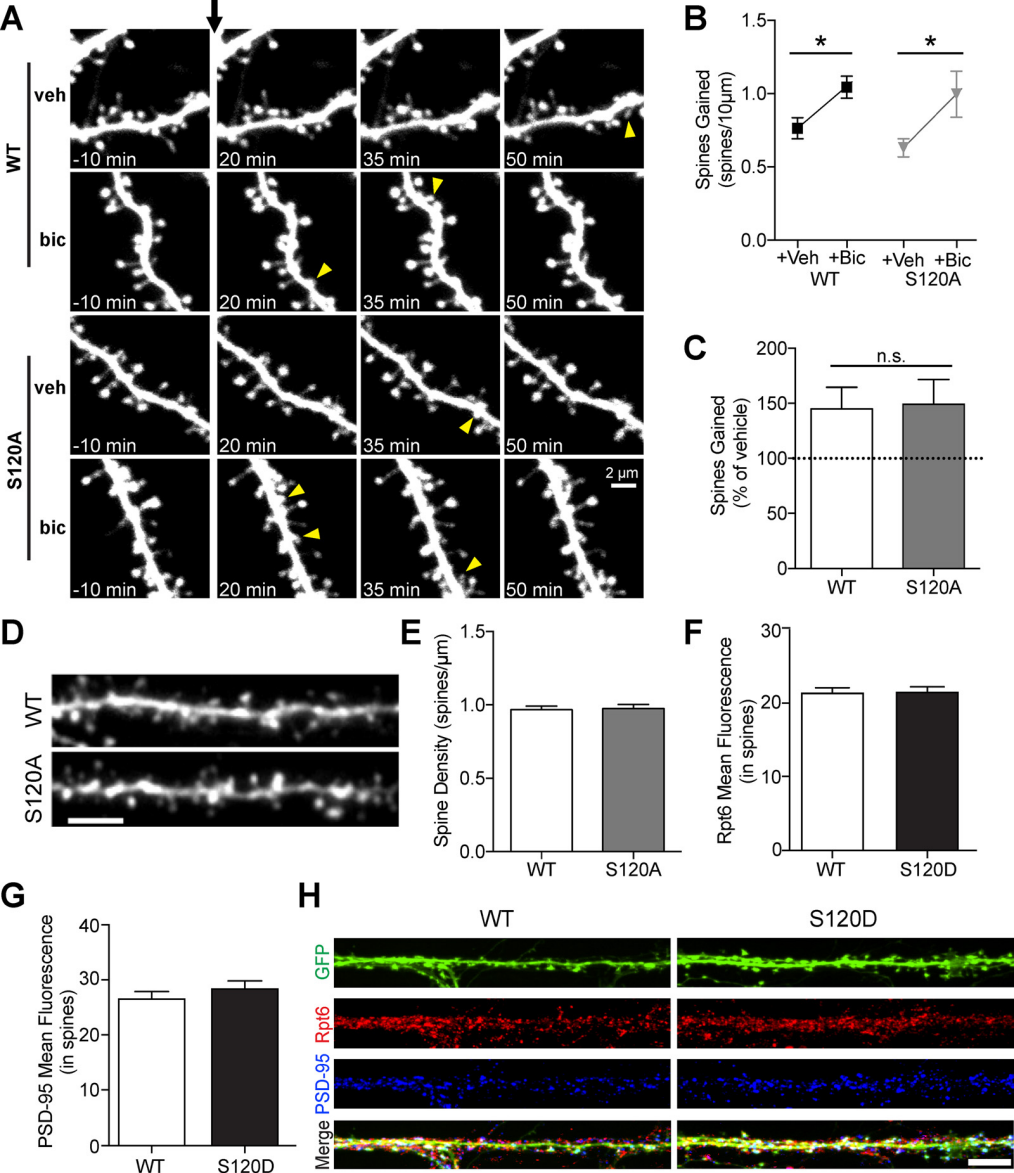
Spine outgrowth dynamics and excitatory synaptic structure are not altered in KI mice. ***A***, Images of dendrites from EGFP-expressing CA1 pyramidal neurons from WT and S120A animals at 7–9 DIV before and after the addition of vehicle (veh) or bicuculline (bic) (30 μm) at *t* = 0 (black arrow). Yellow arrowheads indicate new spines. ***B***, Quantification of number of spines gained per 10 μm with addition of vehicle or bicuculline, in WT (*n* = 6 and 6 neurons, respectively, with one neuron per slice) and S120A (*n* = 7 and 6 neurons, respectively, with one neuron per slice). Bicuculline induced significantly more spine outgrowth than vehicle in both WT and S120A neurons (*p* = 0.02 and *p* = 0.04, respectively), but no significant difference was observed in bic-induced outgrowth between WT and S120A (*p* = 0.79 with *t* test; ANOVA of interaction, *F*_(1,21)_ = 0.197, *p* = 0.66). ***C***, Quantification of normalized spine gain (% of vehicle) in neurons from WT and S120A slices, illustrating similar bic-induced spine outgrowth in both genotypes (*p* = 0.89 with *t* test). ***D***, Representative proximal dendritic segments from hippocampal pyramidal neurons in fixed slices obtained from WT and homozygous S120A mutant littermates, after filling by targeted microinjection of Alexa Fluor 594 Hydrazide. ***E***, Basal spine density quantified by counting the individual number of spine heads on 20-μm segments of secondary dendritic branches in 63× confocal stacks (WT: *n* = 56 segments from 12 cells; S120A: *n* = 51 segments from 10 cells), showing no significant difference in density (*p* = 0.83). ***F***, Quantification of mean Rpt6 fluorescence within dendritic spines of cultured WT and S120D neurons, identified using the GFP channel (*p* = 0.90; *n* = 19 and 20 dendrites from 10 neurons each). ***G***, Quantification of mean PSD-95 fluorescence within spines (*p* = 0.34; *n* = 19 and 20 dendrites from 10 neurons each). ***H***, Example z-stacked immunofluorescent images of GFP-filled (*S. virus*) cultured hippocampal neurons (DIV21–DIV24) from WT and S120D mice stained with Rpt6 (red) and PSD-95 (blue) antibodies. Scale bar: 5 μm. *p < 0.05; n.s. = not significant (*p* > 0.05).

Proteasome function is critical in the maintenance of synaptic plasticity and spine outgrowth. We previously showed that regulation of proteasome function via Rpt6 phosphorylation controls the formation of new spines in response to increased neuronal activity (Hamilton et al., 2012). Furthermore, we found that increasing synaptic activity also augments Rpt6 S120 phosphorylation and proteasome peptidase activity (Djakovic et al., 2009, 2012). Therefore, we wanted to investigate whether the Rpt6 S120A KI mutant that cannot be phosphorylated displays any alteration in activity-induced formation of new spines. Hippocampal pyramidal neurons in organotypic slice cultures of WT and litter-matched homozygous S120A mutants were transfected with EGFP and imaged over time using a two-photon microscope. Dendrites of EGFP-expressing CA1 neurons were imaged at 15 min intervals before and after being treated with either vehicle or 30 μm bicuculline (Fig. 3*A*). Spines were counted and compared with their vehicle controls. We found no difference in the basal spine density measured before treatment (*p* = 0.84, *n* = 6 cells each for WT and S120A). We also observed no significant changes in the overall formation of new spines under basal (vehicle (veh)-treated) conditions or following increased activity (bicuculline (bic)-treated; *p* = 0.89, *n* = 6 cells for both WT and S120A), and bicuculline successfully induced significant spine outgrowth in both genotypes (*p* = 0.02 and *p* = 0.04 for WT and S120A, respectively; Fig. 3*B*,*C*). To further support this finding, we prepared sections of fixed brains from adolescent WT and S120A mice, microinjected CA1 neurons with Alexa Fluor 594 fluorescent dye, and counted the individual number of spine heads on 20-μm segments of secondary dendritic branches using confocal microscopy (Fig. 3*D*,*E*). We found that there was no statistically significant difference between the WT control and S120A mice (*p* = 0.83, *n* = 56 dendrites for WT and *n* = 51 dendrites for S120A), indicating that dendritic spines in the hippocampus develop normally in the absence of S120 phosphorylation dynamics.

We previously reported using ectopically expressed S120 mutants suggested that proteasome distribution in dendrites is affected by the phospho-status of Rpt6 (Djakovic et al., 2012). To directly test this with our KI mice, we generated dissociated hippocampal cultures from WT and S120D mouse pups and used immunostaining to assess the distribution of total Rpt6 and the postsynaptic protein PSD-95, which serves as a marker of excitatory synapses. We observed no differences in Rpt6 (*p* = 0.90) and PSD-95 fluorescence within spines (*p* = 0.34, *n* = 19 and 20 dendrites from WT and S120D cultures, respectively), suggesting that excitatory synapses have formed normally in mutant mice and that the phospho-mimetic status of Rpt6 in S120D mice does not affect overall proteasome distribution in dendrites (Fig. 3*F–H*). Additionally, colocalization of Rpt6 and PSD-95 was identical between the two genotypes, highlighting that Rpt6 presence at excitatory synapses is unchanged (Pearson’s correlation coefficient in WT vs S120D, *p* = 0.50).

### Learning and memory are unaltered in Rpt6 S120A and S120D KI mice

We next sought to evaluate the impact of our KI mutations on learning and memory, because of previous studies linking S120 phosphorylation by CaMKII to long-term memory formation (Jarome et al., 2013). First, to ensure that our mutations did not induce any abnormal anxiety-related behaviors or locomotor deficits that may affect our interpretation of other behavioral data, we assessed these mutants using an elevated plus maze paradigm. S120A (*n* = 8) and WT (*n* = 9) mice did not show any difference in total distance traveled during this test (*p* = 0.16), nor did S120D (*n* = 14) and their WT controls (*n* = 13; *p* = 0.80; Extended Data Fig. 4-1*A*,*B*). Percentage of time spent in the open and closed arms did not differ between WT and S120A mice (*p* = 0.82 and *p* = 0.08, open and closed, respectively), nor did it differ between WT and S120D mice (*p* = 0.17 and *p* = 0.30, open and closed respectively; Extended Data Fig. 4-1*C*,*D*). Thus, these KI mice show normal behavior overall and do not display any apparent anxiety-related phenotypes.

**Figure 4.**
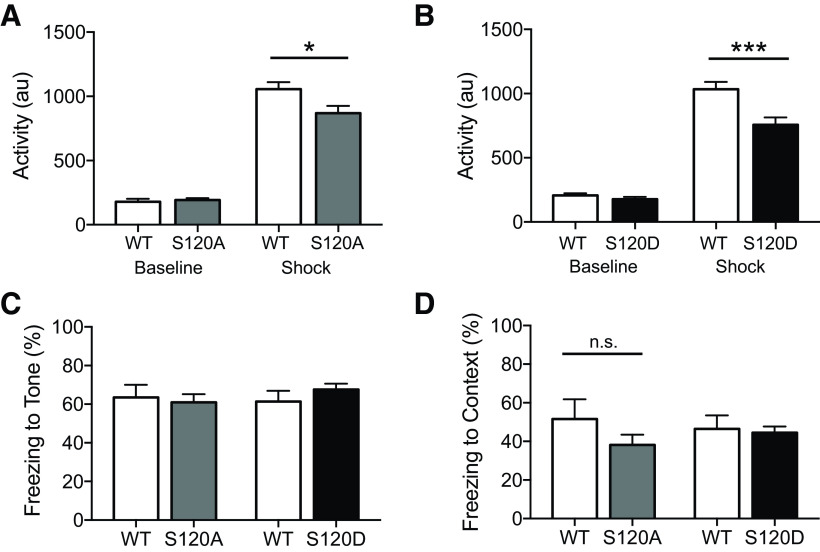
Contextual and cued fear conditioning are unaltered in Rpt6 S120A and S120D KI mice. ***A***, Activity during a 2-min baseline and activity during a 2-s shock in WT (*n* = 8) and S120A (*n* = 16) mice, demonstrating normal baseline activity (*p* = 0.62) but slightly attenuated shock reactivity (ANOVA, *F*_(1,22)_ = 4.33, *p* < 0.05) in S120A mice. ***B***, Same as ***A***, but for WT (*n* = 13) and S120D (*n* = 21), depicting similar baseline activity (*p* = 0.27) but reduced shock reactivity (ANOVA, *F*_(1,32)_ = 10.48, *p* < 0.005). ***C***, Percent time spent freezing to three sequential tone presentations in a novel context, during post-training (24 h) test; S120A and S120D KI mice show similar amounts of freezing compared with respective WT mice (*p* = 0.21, *p* = 0.28, respectively). ***D***, Percent time spent freezing to initial training context during 5 min test, 24 h after training; S120A and S120D mice show normal amounts of freezing (*p* = 0.73, *p* = 0.77, respectively). *p < 0.05; ***p < 0.001; n.s. = not significant (*p* > 0.05).

10.1523/ENEURO.0073-20.2021.f4-1 Extended Data Figure 4-1 Performance on the elevated plus maze was not impaired in S120 KI mice. ***A***, Total distance travelled during elevated plus maze assay, demonstrating no differences between WT (*n* = 9) and S120A (*n* = 8) mice (*p* = 0.16, *t* test). ***B***, Same as ***A***, for WT (*n* = 13) and S120D (*n* = 14; *p* = 0.80, *t* test). ***C***, Percent time spent in each arm of the elevated plus maze (open vs closed) did not differ between S120A (*n* = 8) and WT (*n* = 9) mice (*p* = 0.82 and *p* = 0.08, open and closed, respectively, *post hoc* Bonferroni). ***D***, Same as ***C***, for S120D (*n* = 14) and WT (*n* = 13; *p* = 0.17 and *p* = 0.30, open and closed, respectively, *post hoc* Bonferroni). Download Figure 4-1, PDF file.

The effects of blocking and mimicking Rpt6 phosphorylation at S120 on long-term associative memory were examined using Pavlovian cued and contextual fear conditioning. Baseline activity, measured during the first 2 min of training, did not differ between S120A (*n* = 16) and WT (*n* = 8) mice (*F*_(1,22)_ = 0.25, *p* = 0.62; Fig. 4*A*). However, shock reactivity in S120A mice was slightly dampened (*F*_(1,22)_ = 4.33, *p* = 0.049; Fig. 4*A*). The difference in shock reactivity did not seem to influence memory for the task. When assessed 24 h later, we did not observe any differences in cued (*F*_(1,22)_ = 0.119, *p* = 0.73) nor contextual (*F*_(1,22)_ = 1.645, *p* = 0.21) memory (Fig. 4*C*,*D*). Similarly, S120D (*n* = 21) and WT (*n* = 13) mice displayed no difference in baseline activity (*F*_(1,32)_ = 1.25, *p* = 0.27), but we observed a reduction in shock reactivity (*F*_(1,32)_ = 10.48, *p* < 0.005; Fig. 4*B*). However, this difference once again did not affect learning and memory in this task, as both cued (*F*_(1,32)_ = 1.20, *p* = 0.28) and contextual (*F*_(1,32)_ = 0.087, *p* = 0.77) fear memory were intact in the KI mice (Fig. 4*C*,*D*).

## Discussion

In an effort to further understand the importance of proteasome phosphorylation and its functional relevance at synapses, we created novel mutant mouse lines which constitutively block or mimic phosphorylation of Rpt6 at S120. The impetus to create these mice stemmed from several bodies of work from our and other laboratories which describe how the 26S proteasome is regulated in neurons by synaptic activity. In 2006, Bingol and Schuman provided the first evidence that the 26S proteasome may play an instructive role at synapses in work that described the activity-dependent and NMDA receptor-dependent translocation and sequestration of proteasomes at synapses (Bingol and Schuman, 2006). In addition, we found that neuronal activity regulates proteasome activity by a mechanism involving CaMKIIα-mediated phosphorylation of Rpt6 at S120 (Djakovic et al., 2009, 2012). Subsequently, it was shown that autophosphorylated CaMKIIα acts as a scaffold for proteasomes in dendritic spines (Bingol et al., 2010). In further investigations, we showed that homeostatic scaling of synaptic strength induced by chronic application of bicuculline or tetrodotoxin is both mimicked and occluded by altered Rpt6 phosphorylation (Djakovic et al., 2012). Furthermore, we demonstrated that the acute inhibition of the proteasome or expression of Rpt6 S120A phospho-dead mutant blocks activity-dependent new spine generation (Hamilton et al., 2012). Collectively, these data suggested that CaMKIIα-dependent phosphorylation of Rpt6 at S120 may be an important regulatory mechanism for proteasome-dependent remodeling of synapses.

We first evaluated proteasome peptidase and ATPase activity of intact 26S proteasomes purified from Rpt6 WT, S120A, and S120D mutant mice. Compared with proteasomes purified from WT mice, we found peptidase activity and ATPase activity of proteasomes purified from Rpt6 S120A and S120D mutant to be decreased and increased, respectively, which corroborates previous findings with ectopically expressed Rpt6 S120A and S120D mutants (Djakovic et al., 2009). Unlike proteasome inhibitors, these mutations only alter the activities of the proteasome by ∼15–20%. To determine the functional relevance of these mutations on synapses we evaluated basal synaptic transmission, LTP, spine density and new spine growth, and fear memory. Unlike the striking differences we observed when Rpt6 S120A or S120D mutants are ectopically expressed in neurons (Djakovic et al., 2012; Hamilton et al., 2012), we observed no significant differences in the Rpt6 S120A and S120D KI mice.

Previous work suggested an important role for proteasome-dependent protein degradation in learning and memory. Early studies revealed impairments in one-trial avoidance learning, spatial learning, and aversive taste memory formation on treatment with proteasome inhibitors (Lopez-Salon et al., 2001; Artinian et al., 2008; Rodriguez-Ortiz et al., 2011). Supporting a specific role for S120 phosphorylation in learning, it was observed that fear conditioning transiently increases phosphorylation by CaMKII to stimulate proteasome activity (Jarome et al., 2013) and that this CaMKII-based mechanism of proteasomal regulation may be essential for memory reconsolidation (Jarome et al., 2016). In light of this, the lack of behavioral impairments in our S120 KI mice is somewhat surprising. This either suggests that this particular site is not as essential as previously thought, or that alternate mechanisms are able to differently regulate proteasomal activity to achieve the same end result.

While numerous mechanisms of regulation of the proteasome and its function have been discovered and well characterized, our results demonstrate that Rpt6 S120 phosphorylation dynamics may not play essential roles in the brain. This could be because of an ability of neurons to compensate for minor alterations in proteasome function using either alternative mechanisms of protein degradation, such as lysosomal degradation, or by simply changing transcription/translation rates. Recent work has illustrated the interplay and feedback loops between the UPS and the lysosome system, often showing that when one pathway is inhibited, the other is upregulated to compensate for the disturbance. Specifically, activation of mTORC1, which inhibits lysosomal degradation, has been shown to increase the number of intact and active proteasomes through the activation of NRF1 (Zhang et al., 2014; Zhang and Manning, 2015). In light of this, it is possible that the substrates ordinarily degraded by proteasomes are instead being processed by lysosomes in our mutant mice, which would hide the functional effects of our mutation. Indeed, lysosomes can be dynamically recruited in neurons, and could be poised to compensate for a lack of proteasome recruitment or stimulation (Goo et al., 2017). Future studies are needed to evaluate the careful interplay between these two degradation systems in neurons.

Additionally, it has been shown that certain transcription factors for proteasomal subunits are also substrates of the proteasome, creating a feedback loop where inhibition increases the transcription/translation of proteasome subunits, leading to an increase in active holoenzymes (Xie and Varshavsky, 2001). Interestingly, some of these compensation mechanisms are only observed under low concentrations of proteasome inhibitors, and are absent under high levels of inhibition (Sha and Goldberg, 2014), indicating specialized compensatory mechanisms for minor alterations in activity. Separate from production of proteasomes themselves, it is also possible that the neuronal substrates of proteasomes experience a compensatory change in synthesis. For example, synaptic proteins that would be targeted by proteasomes may be degraded less rapidly or dynamically, but protein translation could be altered concomitantly to make up for this deficit, hiding the true effect of the Rpt6 mutations. Thus, proteins may be able to reach an ideal homeostasis even when degradation is perturbed, through careful orchestration of protein synthesis and degradation mechanisms.

While several other proteasome subunits have been shown to be phosphorylated (Wang et al., 2007; Murata et al., 2009; Tomko and Hochstrasser, 2013; Schmidt and Finley, 2014), the functional relevance of these modifications on proteasome function remains poorly understood. Recently, the cell cycle-dependent phosphorylation of Rpt3 at Thr25 was shown to be important for cell cycle progression and tumorigenesis (Guo et al., 2016), which supports the idea that single site phosphorylation events are clearly important and perhaps rate-limiting on specific proteasome-dependent biological processes. Given that our study focuses only on a single site on Rpt6, we may be missing other key sites of proteasome regulation that could allow proteasomes to be dynamically regulated in neurons.

Although altered Rpt6 S120 phosphorylation dynamics did not affect basal synaptic transmission, LTP, spine dynamics, and fear memory, this does not rule out that there exist biological pathways where downstream effects are dependent on small changes in proteasome activity directly linked to phosphorylation of Rpt6. For instance, our laboratory has recently discovered that behavioral sensitization to cocaine is highly dependent on Rpt6 phosphorylation by CaMKIIα after cocaine administration. In that study, it was shown that cocaine increases CaMKIIα-dependent phosphorylation of Rpt6, and concomitantly increases proteasome activity in the nucleus accumbens and in the prefrontal cortex. In contrast, cocaine does not increase proteasome activity in Rpt6 S120D mutants, and strikingly, there is a complete absence of cocaine-induced locomotor sensitization in the Rpt6 S120D mice (Gonzales et al., 2018), which suggests a critical role for Rpt6 S120 phosphorylation and proteasome function in the regulation of cocaine-induced behavioral plasticity. Furthermore, blocking phosphorylation of Rpt6 at S120 appears to sensitize both yeast and mice to pathogenic effects of Huntington protein aggregation, suggesting that Rpt6 S120 phosphorylation might be engaged under various forms of stress (Lin et al., 2013; Marquez-Lona et al., 2017). It is therefore plausible that Rpt6 phosphorylation could be important and perhaps rate-limiting for proteasome function in other biological pathways in the CNS yet to be identified, or perhaps in specific cell types under conditions that require a high degree of rapid protein turnover. The experiments in the current study focus largely on the hippocampus, specifically on function and plasticity within the Schaffer collateral circuit and on learning paradigms that largely rely on the hippocampus, such as contextual fear memory. It is possible that the hippocampus is robust to our perturbations because of the existence of aforementioned compensatory mechanisms, but other brain regions may be more vulnerable. The impairment in cocaine sensitization in S120D mutants could suggest that the nucleus accumbens is more sensitive to impairments in proteasome dynamics (Gonzales et al., 2018), perhaps because of a higher need for careful control of synaptic protein homeostasis or a lack of effective compensation through changes in synthesis or other degradation pathways. Future studies may identify other brain circuits that similarly rely on proteasome regulation. It is also possible that other forms of plasticity within the hippocampus could be more reliant on Rpt6 phosphorylation, or that very long-lasting (>1 h) LTP requires these dynamics.

Our study suggests that the elimination of phosphorylation dynamics at S120 of Rpt6 fails to have a substantial impact on key forms of learning and synaptic plasticity. These results are surprising in light of the wealth of evidence for this particular site in the control of proteasomes in dendrites, and potentially complicate the current understanding of how proteasomal degradation operates at synapses to carefully control protein homeostasis. We cannot conclude that this site is completely unnecessary for such regulation; our results instead imply that individual neurons and brain circuits have the immense capacity to compensate for deficiencies in degradation. Neurons likely have multiple biological mechanisms that can be employed and manipulated to enable robustness to otherwise deleterious perturbations.

## Synthesis

Reviewing Editor: Bong-Kiun Kaang, Seoul National University Decisions are customarily a result of the Reviewing Editor and the peer reviewers coming together and discussing their recommendations until a consensus is reached. When revisions are invited, a fact-based synthesis statement explaining their decision and outlining what is needed to prepare a revision will be listed below. The following reviewer(s) agreed to reveal their identity: Linda Dansereau.

The authors addressed the reviewers’concerns sufficiently.

Both reviewers also agree that the extended data figures 3-1 and 3-2 could be included in figure 3.
