# Supplementary figures and images for: Altered Phosphorylation of the Proteasome Subunit Rpt6 Has Minimal Impact on Synaptic Plasticity and Learning
Source: eNeuro. 2021 May 4;8(3):ENEURO.0073-20.2021. doi: 10.1523/ENEURO.0073-20.2021 (PMC8116113; doi:10.1523/ENEURO.0073-20.2021)

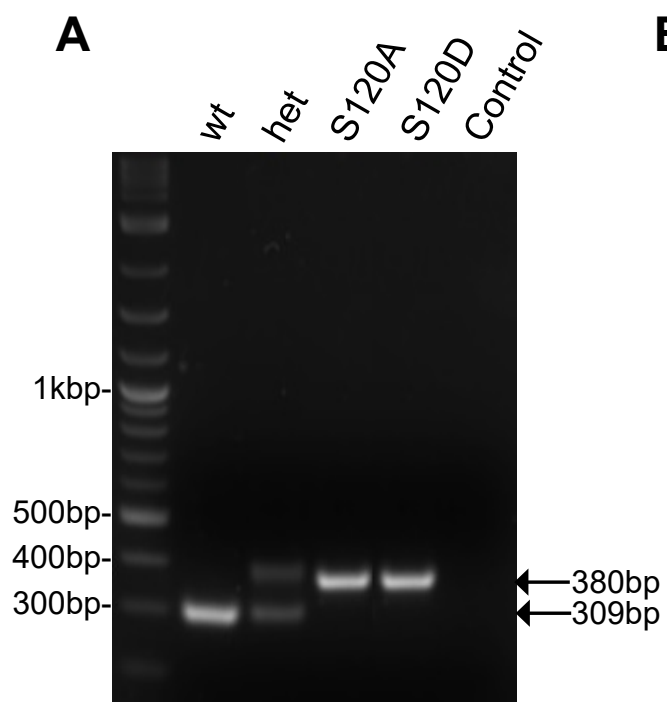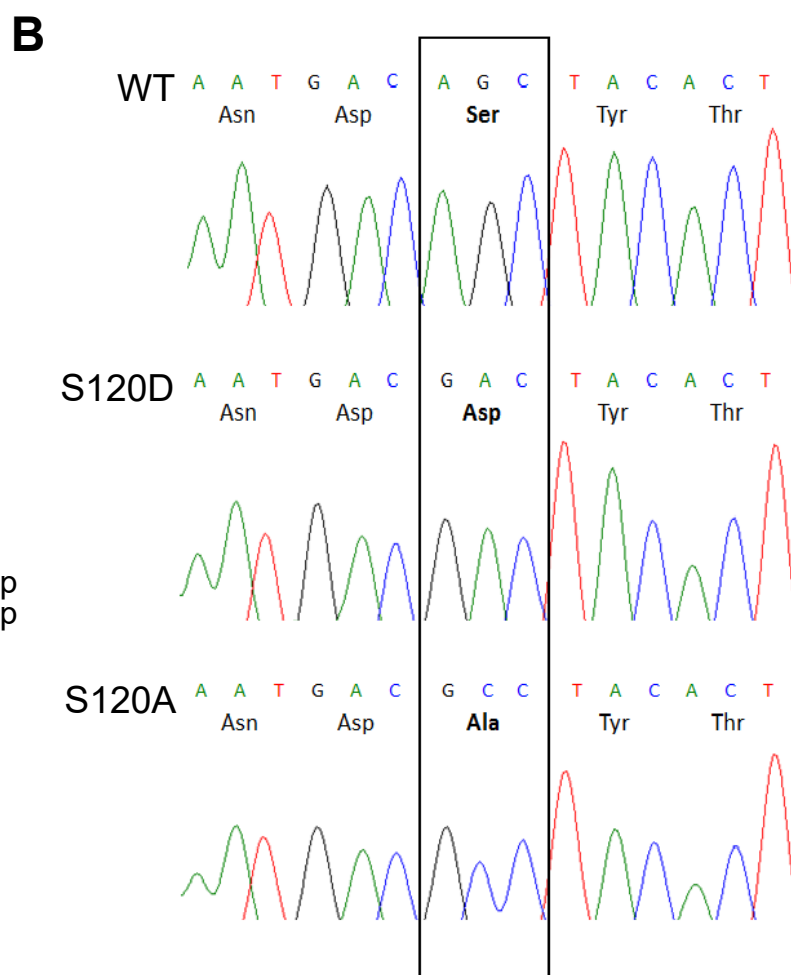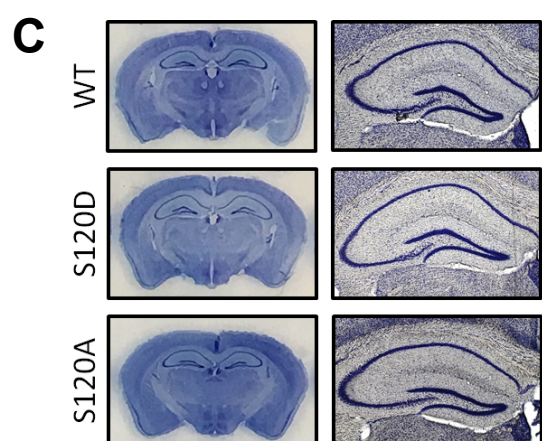

Supplement: Extended Data Figure 1-1 — Generation of Rpt6 S120A and S120D KI mice. A, The original targeting strategy for Rpt6 S120D mutant mice was previously described in Gonzales et al. (2018). Rpt6 S120A mutant mice were made at the same time using the same targeting strategy to create the phospho-ablated mutant. Codon 120 in exon 5 of the PSMC5 (Rpt6) gene was mutated from AGC to GCC (S120 to ala; Gonzales et al., 2018; see also Materials and Methods). A, Tail genomic DNA was analyzed by PCR screening for genotyping and to verify deletion of the Neo cassette. B, Representative electropherograms confirming the presence of the mutation in homozygous Rpt6 S120A and S120D mutant male mice. C, Representative images of Nissl-stained fixed whole-brain coronal sections (with higher magnification of the hippocampus) of 60-d-old mice. Download Figure 1-1, PDF file. [file enu-eN-NWR-0073-20-s01.pdf]

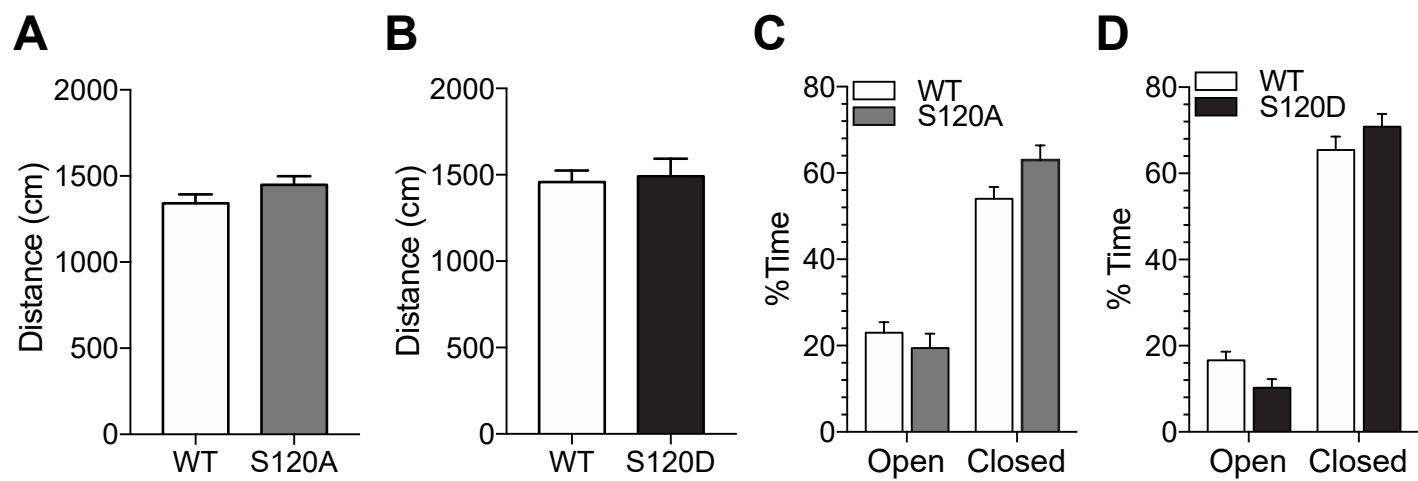

Supplement: Extended Data Figure 4-1 — Performance on the elevated plus maze was not impaired in S120 KI mice. A, Total distance travelled during elevated plus maze assay, demonstrating no differences between WT (n = 9) and S120A (n = 8) mice (p = 0.16, t test). B, Same as A, for WT (n = 13) and S120D (n = 14; p = 0.80, t test). C, Percent time spent in each arm of the elevated plus maze (open vs closed) did not differ between S120A (n = 8) and WT (n = 9) mice (p = 0.82 and p = 0.08, open and closed, respectively, post hoc Bonferroni). D, Same as C, for S120D (n = 14) and WT (n = 13; p = 0.17 and p = 0.30, open and closed, respectively, post hoc Bonferroni). Download Figure 4-1, PDF file. [file enu-eN-NWR-0073-20-s02.pdf]
